# Socioeconomic variation in incidence of primary and secondary major cardiovascular disease events: an Australian population-based prospective cohort study

**DOI:** 10.1186/s12939-016-0471-0

**Published:** 2016-11-21

**Authors:** Rosemary J. Korda, Kay Soga, Grace Joshy, Bianca Calabria, John Attia, Deborah Wong, Emily Banks

**Affiliations:** 1 National Centre for Epidemiology and Population Health, Research School of Population Health, Australian National University, Canberra, ACT Australia; 2 National Drug and Alcohol Research Centre, UNSW Australia, Sydney, NSW Australia; 3 Centre for Clinical Epidemiology and Biostatistics, School of Medicine and Public Health, The University of Newcastle and Hunter Medical Research Institute, Newcastle, NSW Australia; 4 The Sax Institute, Sydney, NSW Australia

**Keywords:** Cardiovascular diseases, Incidence, Socioeconomic factors, Education, Disadvantage, Income, Health status disparities, Inequalities, Cohort, Australia

## Abstract

**Background:**

Cardiovascular disease (CVD) disproportionately affects disadvantaged people, but reliable quantitative evidence on socioeconomic variation in CVD incidence in Australia is lacking. This study aimed to quantify socioeconomic variation in rates of primary and secondary CVD events in mid-age and older Australians.

**Methods:**

Baseline data (2006–2009) from the 45 and Up Study, an Australian cohort involving 267,153 men and women aged ≥ 45, were linked to hospital and death data (to December 2013). Outcomes comprised first event – death or hospital admission – for major CVD combined, as well as myocardial infarction and stroke, in those with and without prior CVD (secondary and primary events, respectively). Cox regression estimated hazard ratios (HRs) for each outcome in relation to education (and income and area-level disadvantage), separately by age group (45–64, 65–79, and ≥ 80 years), adjusting for age and sex, and additional sociodemographic factors.

**Results:**

There were 18,207 primary major CVD events over 1,144,845 years of follow-up (15.9/1000 person-years), and 20,048 secondary events over 260,357 years (77.0/1000 person-years). For both primary and secondary events, incidence increased with decreasing education, with the absolute difference between education groups largest for secondary events. Age-sex adjusted hazard ratios were highest in the 45-64 years group: for major CVDs, HR (no qualifications vs university degree) = 1.62 (95% CI: 1.49–1.77) for primary events, and HR = 1.49 (1.34–1.65) for secondary events; myocardial infarction HR = 2.31 (1.87–2.85) and HR = 2.57 (1.90–3.47) respectively; stroke HR = 1.48 (1.16–1.87) and HR = 1.97 (1.42–2.74) respectively. Similar but attenuated results were seen in older age groups, and with income. For area-level disadvantage, CVD gradients were weak and non-significant in older people (> 64 years).

**Conclusions:**

Individual-level data are important for quantifying socioeconomic variation in CVD incidence, which is shown to be substantial among both those with and without prior CVD. Findings reinforce the opportunity for, and importance of, primary and secondary prevention and treatment in reducing socioeconomic variation in CVD and consequently the overall burden of CVD morbidity and mortality in Australia.

**Electronic supplementary material:**

The online version of this article (doi:10.1186/s12939-016-0471-0) contains supplementary material, which is available to authorized users.

## Background

Cardiovascular disease (CVD) is the leading cause of death and disability globally [1, 2]. In Australia, although CVD mortality has decreased around 70% since the early 1980s [3], more people die from ischaemic heart disease than any other disease, followed closely by stroke [4], and CVD accounts for the greatest health care expenditure of any major disease group [5]. Around one in five Australians aged 45–74 are estimated to be at high absolute CVD risk — just over half of these have high primary risk, and the remainder have a history of prior CVD and hence are at high risk of a secondary event [6]. Despite this high background level of risk, a large proportion of CVD events can be prevented using population- and individual-level interventions and there remains substantial potential for further reductions in CVD incidence and mortality.

The potential for CVD prevention is likely to be greatest in the most disadvantaged groups within the population, given that the CVD burden is highest in these groups [7]. Nevertheless, there is a lack of reliable quantitative evidence on socioeconomic variation in the incidence of CVD in Australia. In this country, aggregate CVD hospital admissions and mortality data are typically used to report on variation in CVD, with inequalities described in relation to area-level disadvantage [7–10]. However, these data do not allow estimation of CVD incidence, and they will necessarily underestimate socioeconomic variation in CVD events. In contrast, population-based prospective cohort studies can quantify variation in incidence by incorporating individual-level socioeconomic factors and tracking CVD events (both fatal and non-fatal) in individuals. Studies of this type undertaken in high-income countries other than Australia generally report higher incidence of primary (incident) CVD, usually myocardial infraction or stroke, among people of lower socioeconomic position (SEP) [10–29]. However, while the relationships between SEP and CVD outcomes are broadly similar across industrialised countries and may be similar in Australia, local contemporary data are important for quantifying the magnitude of the problem. This evidence is critical to prioritising and targeting interventions, and for evaluating progress. We could identify only two population-based prospective studies using Australian data. One study of mid-age women found decreasing incidence of self-reported stroke with increasing education, and a pooled cohort study using linked data (Australia and New Zealand (NZ) combined), reported increasing event rates (primary and secondary combined) with decreasing education for total CVD and coronary heart disease, but not for stroke [30].

World-wide, there is scant evidence on socioeconomic variation in incidence of secondary CVD events (i.e. in those with existing CVD or history of a prior event) [31]. We could not identify any Australian studies on this. The distinction between primary (incident) and secondary events is important given the much larger absolute risk of a CVD event in those with prior CVD and hence potentially greater absolute benefits from intervention. In addition, approaches to prevention and treatment, and methodological implications, for these outcome types differ. Quantification of such variation necessarily requires individual-level longitudinal data on both non-fatal and fatal specific CVD endpoints; information on prior CVD history; and individual-level rather than area-based measures of SEP, with studies based on the latter likely to underestimate variation [32].

The aim of this study was to use large-scale individual-level linked survey and administrative data to quantify individual-level socioeconomic variation in rates of primary and secondary CVD events, in a population-based cohort of mid-age and older Australians.

## Methods

### Data

We used data from the Sax Institute’s 45 and Up Study, an Australian cohort involving 267,153 men and women aged 45 and over from New South Wales (NSW), Australia. Participants in the Study were randomly sampled from the database of Australia’s universal health insurance provider, Medicare Australia, with over-sampling by a factor of two, of individuals aged 80 years and over and people resident in rural areas. Around 10% of the entire NSW population aged 45 and over were included in the sample. Participants joined the Study by completing a baseline questionnaire (between Jan 2006 and April 2009) and giving signed consent for follow-up and linkage of their information to a range of health databases. The Study is described in detail elsewhere [33], and questionnaires can be viewed online [34].

Baseline survey data from the participants were linked to hospital data from the NSW Admitted Patient Data Collection (APDC, 1 July 2000 to 31 December 2013), data on date of death from the NSW Registry of Births, Deaths and Marriages (1 January 2006 to 31 December 2013), and data on causes of death from the Cause of Death Unit Record File (1 January 2006 to 31 December 2013). The APDC includes records of all hospitalisations in NSW, dates of admission and discharge and reasons for admission. Each record in APDC contains up to 51 diagnosis codes using the International Statistical Classification of Diseases and Related Health Problems, Tenth Revision, Australian Modification (ICD-10-AM) codes and up to 50 procedure codes using the Australian Classification of Health Interventions (ACHI) codes. The Cause of Death Unit Record File includes primary causes of death and up to 20 additional causes using ICD-10-AM. Data were linked probabilistically by the Centre for Health Record linkage using personal information (including full name, date of birth, sex and address). Over the relatively short follow-up period, a small but unknown number of participants are likely to have moved out of NSW. Although hospitalisations occurring in neighbouring states would not be captured, these are estimated to make up fewer than 2% of admissions in NSW residents. Hence, follow-up for hospitalisations is considered to be ~98% complete among those continuing to reside in NSW. Quality assurance data on the data linkage show false positive and negative rates of < 0.5% and < 0.1%, respectively.

### Outcomes

The main outcome was a major CVD event: a composite endpoint of fatal or non-fatal major CVD, ascertained through first hospital admission for major CVD, or death due to CVD, following recruitment into the study. We defined major CVD as a sub-group of circulatory diseases that have a significant atherosclerotic or arteriovenous thromboembolic component, based on a combination of diagnosis codes from ICD-10-AM and CVD-related intervention procedure codes from the 5^th^ to 7^th^ editions of ACHI [35]. In addition, we separately examined two common CVD subtypes: myocardial infarction (ICD-10-AM codes: I21 and I22) and stroke (intracerebral haemorrhage, infarction or transient ischaemic attack, ICD-10-AM codes: I61, I63, I64, and G45). In a supplementary analysis we also report results for ischaemic heart disease combined (ICD-10-AM codes: I20–I25) for comparison with other published results. We ascertained outcomes using the primary diagnosis code field (and procedure fields) of the APDC, and the primary cause of death code field of the Cause of Death Unit Record File.

In order to distinguish primary from secondary CVD events, we analysed outcomes separately in those with and without prior history of CVD. Prior CVD was defined as self-reported heart disease, stroke, or blood clot (thrombosis) on the baseline questionnaire, and/or hospital admission for major CVD ascertained from the 51 diagnosis code fields and the 50 procedure code fields of APDC in the 6 years prior to entering the study.

For each analysis, participants contributed person-years from recruitment date to the outcome of interest (first major CVD/myocardial infarction/stroke/ischaemic heart disease hospital admission or death), death from any cause, or end of follow up (31 December 2013), whichever was the earliest.

### Main exposure: socioeconomic position

Socioeconomic position was based on education attainment, as well as two supplementary socioeconomic exposures for comparison—annual household income and area-level disadvantage. Education attainment was used as the primary socioeconomic variable as it is an individual as opposed to area-based measure. In addition, unlike household income, education attainment is a stable indicator of SEP from relatively early in the life course, is unlikely to be subject to reverse causality (i.e. CVD outcomes impacting on SEP), and is considered to be reliably reported with little missing data.

Education attainment was self-reported in defined categories, which were grouped for the analysis: No qualifications (“no school certificate or other qualifications”); certificate/diploma/trade (“school or intermediate certificate or equivalent,” “higher school or leaving certificate or equivalent,” “trade/apprenticeship, e.g. hairdresser, chef,” “certificate/diploma, e.g., child care, technician”); and university degree (“university degree or higher”). Annual household income (from all sources, before tax) was self-reported in six defined brackets, which were grouped for analysis: < $20,000, $20,000- < $40,000, $40,000- < $70,000, ≥ $70,000 and missing. Area-level disadvantage was based on the Australian Bureau of Statistics Index of Relative Socio-economic Disadvantage (IRSD), a measure derived from Census data which summarises socioeconomic disadvantage in a particular area. [36] We categorised the IRSD into population-based quintiles using 2006 Australian Census data, and assigned it to individuals using their postcode of residence.

### Analysis

All analyses were conducted separately in those with and without prior CVD. First, we calculated rates of major CVD in relation to education, separately in males and females. Rates were standardised by age to the 2006 NSW population, in 5-year age groups, using the direct method [37], and rates differences (RD) and rate ratios (RR) were calculated, comparing rates in the lowest education group to those in the highest.

Second, Cox regression was used to estimate hazard ratios (HRs) for each outcome (major CVD/myocardial infarction/stroke/ischaemic heart disease hospital admission or death) in relation to education, with age as the underlying time variable, as a measure of relative differences in outcomes according to SEP. Analyses were performed separately for three age groups (45–64, 65–79, and ≥ 80 years). Model 1 was adjusted for age (as the underlying time variable) and sex. Model 2 was adjusted for age, sex, region of birth (born in Australia/NZ and born in other countries) and region of residence (major cities, inner regional, and outer regional/remote/very remote). Model 3 was adjusted for the same factors as Model 2 and additionally adjusted for private health insurance (hospital/Department of Veterans Affairs concession card and no private health insurance). Participants with missing values for the main SEP measure were dropped from that analysis. Missing values for covariates were included in the models as separate categories. In supplementary analyses, we calculated age-adjusted rates and estimated HRs for major CVD in relation to income and area-based disadvantage (Model 1 only).

Two sets of sensitivity analyses were performed. In the first, we re-defined prior history of CVD to exclude self-reported blood clot (thrombosis) and re-ran the analyses for all outcomes; in the second analyses, we excluded transient ischaemic attack from the definition of stroke.

The proportional hazards assumption was verified using tests based on the Schoenfeld residuals for each model (significance level of 0.0001 was used due to the large sample size). Stratified forms of the models were used where covariates showed non-proportionality of hazards. Tests for linear trend were also performed for each model. All analyses were performed using Stata version 12.

### Results

After excluding individuals with linkage errors (*n* = 196) and those aged less than 45 years at baseline (*n* = 8), the sample included 266,684 participants. Mean age was 63 years (Standard deviation (SD) =11), with 61% aged 45–64 years, 28% aged 65–79 years, and 10% aged 80+ years. Just over half of all participants (54%) were female. Of the total sample, 12% had no qualifications, 65% a certificate, diploma or trade and 23% a university degree, with education levels higher in the younger than older cohorts. One in five people (22%) reported a history of prior CVD, ranging from 12% in those aged 45–64, to 51% in those aged 80 or older. Further sample characteristics are shown in Table 1.

**Table 1:** Characteristics of study participants at baseline

Age group	45-64		65-79		≥80		All age groups	
Mean age ± SD	55 ± 5.41		71 ± 4.26		84 ± 3.54		63 ± 11.17	
	Number	%	Number	%	Number	%	Number	%
All participants	163 660	100	75 940	100	27 084	100	266 684	100
Sex								
Male	70 502	43	39 300	52	13 893	51	123 695	46
Female	93 158	57	36 640	48	13 191	49	142 989	54
Education								
No qualifications	13 690	8	12 320	17	5 225	20	31 235	12
Certificate/diploma/trade	102 243	63	50 004	67	17 231	66	169 478	65
University degree	46 083	28	11 966	16	3 465	13	61 514	23
Annual household income								
< $20,000	18 853	14	23 541	42	10 027	55	52 421	25
$20,000- < $40,000	23 904	18	17 743	31	4 988	27	46 635	22
$40,000- < $70,000	35 401	26	9 567	17	2 128	12	47 096	23
≥$70,000	55 905	42	5 676	10	1 195	7	62 776	30
IRSD socioeconomic quintile								
1 (most disadvantaged)	30 095	18	16 234	21	4 629	17	50 958	19
2	39 896	24	20 384	27	5 975	22	66 255	25
3	33 061	20	14 763	19	4 978	18	52 802	20
4	30 579	19	12 664	17	5 155	19	48 398	18
5 (least disadvantaged)	29 866	18	11 858	16	6 338	23	48 062	18
Region of birth								
Australia/NZ	127 970	79	57 703	77	19 191	72	204 864	77
Other	34 720	21	17 343	23	7 417	28	59 480	23
Region of residence								
Major cities	72 554	44	30 653	40	16 867	62	120 074	45
Inner regional	57 828	35	29 087	38	6 793	25	93 708	35
Outer regional/remote/very remote	33 126	20	16 166	21	3 415	13	52 707	20
Private health insurance								
Yes (hospital/DVA)	111 826	68	45 687	60	16 305	60	173 818	65
No	51 833	32	30 247	40	10 777	40	92 857	35
Prior major CVD								
Yes	19 577	12	24 953	33	13 775	51	58 305	22
No	144 083	88	50 987	67	13 309	49	208 379	78

% show characteristics within a given age group. Denominators of the percentages do not include missing cases. Number of missing cases: education = 4,457 (1.7%); annual household income = 57,756 (21.7%); IRSD socioeconomic quintile = 209 (0.1%); region of birth = 2,340 (0.9%); region of residence = 195 (0.1%); private health insurance = 9 (<0.1%)

There were a total of 38,255 major CVD events over 1,405,202 years of follow-up (median follow-up = 5.37 years), a rate of 27.2 per 1000 person-years. There were 18,207 primary major CVD events (i.e. events in people with no prior CVD) over 1,144,845 years, a rate of 15.9 per 1000 person-years, and 20,048 secondary events (i.e. events in people with prior CVD) over 260,357 years, a rate of 77.0 per 1000 person-years.

**Fig. 1:**
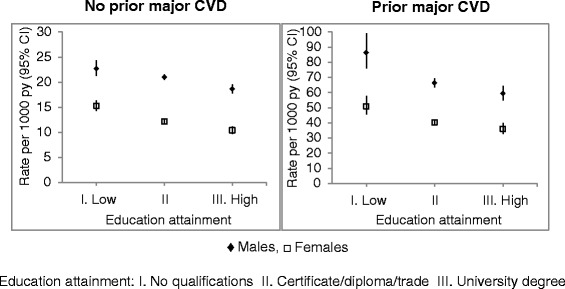
Age-adjusted rates of major cardiovascular disease (CVD) events by education, in those with and without prior CVD

For both primary and secondary major CVD events, age-standardised rates decreased with increasing education, among both males and females (Fig. 1). For primary events, age-standardised rates for males ranged from 18.6 per 1000 person years among those with a university degree to 22.7 per 1000 person years among those with no school qualifications (RD = 4.07; RR = 1.22), with the corresponding rates in females being 10.4 and 15.3 per 1000 person years (RD = 4.86; RR = 1.47). Rate differences were notably higher for secondary events, with age-standardised rates for males ranging from 59.4 per 1000 person years among those with a university degree to 86.4 per 1000 person years among those with no qualifications (RD = 27.0; RR = 1.45); the corresponding rates in females were 36.0 and 50.9 per 1000 person years (RD = 14.9; RR = 1.41).

After adjusting for age and sex (Model 1), HRs increased with increasing education in each age group for both primary and secondary events, as indicated by the tests for trend (Table 2). In the 45–64 years age group, rates were around 50–60% higher among those with no qualifications than among those with a university degree, for both primary (HR = 1.62, 95% CI: 1.49–1.77) and secondary (HR = 1.49; 95% CI: 1.34–1.65) major CVD events. Additional adjustment for region of birth and region of residence (Model 2) and also private health insurance (Model 3) made little difference to the HR estimates (Additional file 1: Table S1). Similar but attenuated results were seen in the older age groups (65–79 and ≥ 80 years) (Table 2).

**Table 2:** Crude rates of major cardiovascular disease (CVD), myocardial infarction and stroke events and adjusted hazard ratios (HR), by education, in those with and without prior CVD

	No prior major CVD			Prior major CVD		
	Events/py^a^	Crude rates^b^	Adjusted HR^c^ (95% CI)	Events/py^a^	Crude rates^b^	Adjusted HR^c^ (95% CI)
*Major CVD*						
45–64 years						
No qualifications	776/62450	12.43	1.62 (1.49–1.77)	644/12269	52.49	1.49 (1.34–1.65)
Cert./diploma/trade	4663/505833	9.22	1.27 (1.20–1.35)	2630/63613	41.34	1.17 (1.08–1.27)
University degree	1669/236864	7.05	1.00	795/22087	35.99	1.00
*p* (test for trend)			< 0.0001			< 0.0001
65–79 years						
No qualifications	1171/41,502	28.22	1.13 (1.04–1.23)	1672/19615	85.24	1.24 (1.15–1.34)
Cert./diploma/trade	4830/178362	27.08	1.11 (1.04–1.18)	5929/72579	81.69	1.17 (1.10–1.24)
University degree	1123/44967	24.97	1.00	1147/15962	71.86	1.00
*p* (test for trend)			0.0032			< 0.0001
≥ 80 years						
No qualifications	744/11810	63.00	1.11 (0.99–1.25)	1338/9213	145.23	1.15 (1.06–1.26)
Cert./diploma/trade	2300/39396	58.38	0.99 (0.90–1.10)	4400/32563	135.12	1.07 (0.99–1.15)
University degree	483/7912	61.05	1.00	871/6666	130.67	1.00
*p* (test for trend)			0.0402			0.0009
*Myocardial infarction*						
45–64 years						
No qualifications	142/64112	2.21	2.31 (1.87–2.85)	103/13973	7.37	2.57 (1.9–3.47)
Cert/diploma/trade	813/516122	1.58	1.68 (1.45–1.95)	351/70833	4.96	1.69 (1.31–2.17)
University degree	227/240648	0.94	1.00	74/24280	3.05	1.00
*p* (test for trend)			<.0001			<.0001
65–79 years						
No qualifications	201/44059	4.56	1.62 (1.3–2.01)	285/23645	12.05	1.79 (1.46–2.20)
Cert./diploma/trade	756/188974	4.00	1.44 (1.2–1.72)	824/87810	9.38	1.36 (1.14–1.63)
University degree	138/47538	2.90	1.00	137/19134	7.16	1.00
*p* (test for trend)			< .0001			< .0001
≥ 80 years						
No qualifications	157/13017	12.06	1.37 (1.05–1.80)	274/11678	23.46	1.38 (1.13–1.68)
Cert./diploma/trade	470/43431	10.82	1.18 (0.93–1.49)	806/40975	19.67	1.14 (0.95–1.35)
University degree	85/8812	9.65	1.00	154/8424	18.28	1.00
*p* (test for trend)			0.0164			0.0010
*Stroke*						
45–64 years						
No qualifications	97/64333	1.51	1.48 (1.16–1.87)	78/14046	5.55	1.97 (1.42–2.74)
Cert./diploma/trade	601/516740	1.16	1.21 (1.03–1.41)	275/70991	3.87	1.38 (1.06–1.81)
University degree	224/240687	0.93	1.00	67/24376	2.75	1.00
*p* (test for trend)			0.0009			< .0001
65–79 years						
No qualifications	203/44046	4.61	1.08 (0.88–1.31)	290/23614	12.28	1.29 (1.07–1.55)
Cert./diploma/trade	846/188872	4.48	1.09 (0.93–1.28)	940/87274	10.77	1.13 (0.96–1.32)
University degree	191/47433	4.03	1.00	182/18963	9.6	1.00
*p* (test for trend)			0.4795			0.0067
≥ 80 years						
No qualifications	189/12917	14.63	1.08 (0.85–1.36)	320/11548	27.71	1.44 (1.18–1.76)
Cert./diploma/trade	604/43098	14.01	1.02 (0.84–1.24)	991/40330	24.57	1.32 (1.11–1.57)
University degree	120/8728	13.75	1.00	151/8347	18.09	1.00
*p* (test for trend)			0.4983			0.0006

^a^Events = incident hospital admission or death and py = person years of follow-up. ^b^Rates are per 1000 person-years. ^c^HRs adjusted for age and sex

Analyses conducted for myocardial infarction only, which accounted for 17% of primary and 16% of secondary major CVD events, obtained similar patterns to analyses for total major CVD events but HRs were substantially higher, in all age groups (Table 2). In the 45–64 years age group, myocardial infarction rates were around two and half times higher among those with no qualifications than among those with a university degree for both primary (HR = 2.31, 95% CI: 1.87–2.85) and secondary (HR = 2.57; 95% CI: 1.90–3.47) events. Even in the older participants, rates were around 40% higher in the least compared to the most educated group (primary event HRs: 1.37, 95% CI: 1.05–1.80; and secondary events: HR = 1.38, 95% CI: 1.13–1.68). Analyses conducted for ischaemic heart disease events combined, which accounted for nearly half of all primary (44%) and secondary (47%) major CVD events, obtained similar results to analyses for total major CVD events (Additional file 2: Table S2).

Patterns for stroke, which accounted for 17% of both primary and secondary major CVD events, were also similar to those for all major CVD although trends were not significant for primary events among the two older age groups (65–79 and ≥ 80 years, Table 2). Hazard ratios were again highest in the 45–64 year age group, with a 50% higher risk of stroke among those with no qualifications compared to those with a university degree, for primary events (HR = 1.48, 95% CI: 1.16–1.87) and a nearly two-fold (100%) greater risk for secondary events (HR = 1.97, 95% CI: 1.42–2.74).

Hazard ratios for the two sets of sensitivity analyses — one excluding self-reported thrombosis from the definition of prior CVD (resulting in 928 events (2.5%) being re-classified as primary rather than secondary), and the other excluding transient ischaemic attack from the definition of the stroke outcome (resulting in 2289 (36%) fewer events) — did not differ materially from those for the main analyses.

Similar relationships between SEP and major CVD incidence were seen when rates were modelled according to annual household income, although SEP trends were not significant in the older age group (≥ 80 years); when area-level disadvantage was the measure of SEP, gradients were weak (45–64 years age group) or non-significant (65–79 and ≥ 80 years age groups) (Additional file 3: Figure S1 and Additional file 4: Table S3).

## Discussion

Large-scale, individual-level prospective data on mid-age and older Australians revealed substantial socioeconomic variation in the incidence of both primary and secondary CVD events, with rates higher among disadvantaged people. For both those with and without prior CVD in age group 45–64, major CVD incidence rates were around 50–75% higher, myocardial incidence around 250% higher and stroke incidence rates around 50–100% higher, among those of low SEP (no educational qualifications) compared to those of high SEP (university degree). Similar but attenuated relative inequalities were seen in the two older age groups (65–79 and ≥ 80); however, when using area-level as a proxy for individual SEP there was no significant socioeconomic variation in these older age groups. The absolute difference in rates between socioeconomic groups was considerably larger for secondary than primary events, with rate differences in overall CVD incidence between the highest and lowest educational groups for secondary major CVD events six-fold higher than for primary major CVD events in males, three-fold higher in females.

While it is difficult to directly compare the magnitude of socioeconomic variation in CVD across studies, that incidence of primary CVD events increased with increasing disadvantage is consistent with international evidence. With few exceptions, (e.g., [30]), prospective cohort studies in high-income countries report higher incidence of ischaemic heart disease and stroke among those of lower SEP [10, 11, 13–26, 28, 29, 31, 38–43]. They are also consistent with a recent large prospective United Kingdom (UK) study, where relative rates of atherosclerotic CVD subtypes, including unstable angina, ischaemic stroke, intracerebral haemorrhage, myocardial infarction, heart failure and peripheral arterial disease, varied inversely with area-level deprivation [43]. Our findings on socioeconomic variation in stroke incidence are also consistent with an Australian prospective study on incident stroke in mid-age Australian women (1996–2008) using self-reported data [44], and with a study based on aggregated stroke registry and census data (1995–2003) [45]. However they differ from a pooled cohort study using Australian/NZ data, which found increasing event rates (primary and secondary combined) with decreasing education for all CVD and coronary heart disease, but not for stroke [30]. These differing findings may not only reflect differences within the Australian and NZ populations but also different methods (definition of endpoints, consideration of prior CVD history), and given the attenuation of risks in older age groups, different age structures across the studies.

To our knowledge, this is the first Australian study to have quantified variation in incidence of secondary CVD events and there are few international studies with which to compare our findings. The magnitude of income-related relative inequalities in rates of incident myocardial infarction reported in a Finnish population-based study were slightly lower than those for incident and subsequent myocardial infarctions combined [39]. Limited evidence also comes from prospective studies on stroke recurrence in several European studies. In a Swedish prospective study (1990–2001), while incident stroke was associated with lower income and occupation in both men and women, recurrent stroke was inversely associated with income only in women [40]; in an Italian study (2001–2004), among those who had survived a first stroke, low SEP (measured at the small area level) was associated with a subsequent admission for stroke (in men) and cardiovascular disease (in women) [46]; a UK population-based study (1995–2004) reported no difference in stroke recurrence between manual and non-manual occupations [47]. Despite the lack of previous evidence for comparison, the higher absolute inequalities for secondary compared to primary CVD events found in this study is consistent with *a priori* expectations, given that the absolute risk of a CVD event is higher amongst those who have had a prior CVD event than among those who have not.

That relative inequalities were greater in mid-age (45–64 years) than older people—across the various outcomes and the different measures of SEP—is in line with previous international evidence [15, 16, 23, 25, 39, 48]. This attenuating RR will occur wherever absolute risk increases with increasing exposure (as is usually the case with age) and RDs remain constant (and in some cases even if RDs rise). This age attenuation in RRs may also reflect the ‘survivor effect’ (whereby the negative effect of low SEP on health means those remaining in the cohort are not a random sample of the population but rather reflect those who are more likely to have survived the effect of low SEP on premature mortality); change in risk actors with age; and/or that the selected SEP variables—education, household income and area-level disadvantage—are less accurate measures of SEP in older than younger people. That socioeconomic variation was not apparent in the two older age groups when area-level was used as the SEP measure is consistent with the expectation that when such measures are used as a proxy for individual SEP there is likely to be considerable misclassification of individuals, and hence, underestimation of socioeconomic variation in the outcome. As has been noted previously, this underestimation is important to take into account when making decisions based on area-level inequality estimates [32].

The strengths of our prospective cohort study include: its large sample size, enabling examination of outcomes separately in relation to CVD subtypes and by age group; independent ascertainment of outcomes, including both fatal and non-fatal endpoints, with virtually complete follow-up through linkage to administrative hospital and death records; and availability of individual-level socio-demographic factors and measures of SEP. However, there are several limitations that should be borne in mind when interpreting the results. First, administrative hospital and death data may not capture all major CVD events, although they are likely to capture the vast majority. Second, data on education and income were self-reported, which means possible misclassification of SEP, although this is likely to be non-differential and, if anything, bias results towards the null; also we did not capture changes over time in SEP. Third, while the 45 and Up cohort are broadly representative of the Australian population in this age group, they are likely to be healthier and have lower hospitalisation and mortality rates than the general population in this age group. Further, consistent with many population-based cohort studies, the design aimed to maximise heterogeneity of exposure and to retain participants rather than provide a sample that is necessarily representative of the general population, and thus caution should be used when interpreting and generalising absolute incidence estimates. However, representativeness is not necessary for reliable estimates of relative rates based on internal comparisons within study populations [49, 50], and the relative inequality estimates, as measured in this study, are assumed to be valid and broadly generalizable, albeit potentially biased toward the null given the likelihood of some non-differential misclassification of SEP and outcome variables. Finally, the study did not set out to attribute causality, hence the minimal adjustment for other explanatory variables in the models. Consequently, some caution should be applied when attributing causality. This caution particularly applies to the findings on variation in secondary events by income due to the possibility of reverse causality (i.e. that a previous CVD event will lead to a loss of income), especially in the 45–64 age group who are of working-age.

## Implications and conclusions

Individual-level longitudinal data on both fatal and non-fatal CVD outcomes are important for quantifying socioeconomic variation in CVD incidence. Based on individual-level SEP measures, socioeconomic variation in CVD incidence is shown to be substantial, not just for primary events, but also for secondary events. Given CVD is largely preventable and socioeconomic variation technically avoidable, our findings suggest large potential for reductions in CVD burden.

While our study did not examine the reasons underlying socioeconomic variation in incidence, it is likely to include variation in behavioural risk factors such as smoking and physical activity, and also health care. The novel finding that the largest socioeconomic differences are among those who have had a prior CVD event — and are therefore likely to be in contact with the health care system — reinforces the opportunity for, and importance of, optimal treatment and secondary prevention.

CVD is a national health priority for the Australian Government. Reducing the socioeconomic variation in incidence of both primary and secondary events should be a target in itself, not only to reduce health inequalities, but as a mechanism for lowering the overall burden of CVD morbidity and mortality in Australia.

## Additional files

Additional file 1: Table S1. Comparison of models for major cardiovascular disease (CVD) events rates by level of education, in those with and without prior CVD. (PDF 185 kb)
Additional file 2: Table S2. Crude rates of ischemic heart disease events and adjusted hazard ratios (HR), by education, in those with and without prior CVD. (PDF 180 kb)
Additional file 3: Figure S1. Age-adjusted rates of major cardiovascular disease (CVD) events by household income and area-level disadvantage, in those with and without prior CVD. (PDF 228 kb)
Additional file 4: Table S3. Crude rates of major cardiovascular disease (CVD) events and adjusted hazard ratios (HR) by household income and area-level disadvantage, in those with and without prior CVD. (PDF 211 kb)

## Abbreviations

ACHI: Australian classification of health interventions
APDC: Admitted patient data collection
CI: Confidence intervals
CVD: Cardiovascular disease
HR: Hazard ratios
ICD-10-AM: International statistical classification of diseases and related health problems, tenth revision, Australian modification
IRSA: Index of Relative Socio-economic Advantage
NSW: New South Wales
NZ: New Zealand
RD: Rate difference
RR: Rate ratio
SD: Standard deviation
SEP: Socioeconomic position
UK: United Kingdom
