# Erratum to: Socioeconomic variation in incidence of primary and secondary major cardiovascular disease events: an Australian population-based prospective cohort study

**DOI:** 10.1186/s12939-016-0502-x

**Published:** 2017-01-12

**Authors:** Rosemary J. Korda, Kay Soga, Grace Joshy, Bianca Calabria, John Attia, Deborah Wong, Emily Banks

**Affiliations:** 1 National Centre for Epidemiology and Population Health, Research School of Population Health, Australian National University, Canberra, ACT Australia; 2 National Drug and Alcohol Research Centre, UNSW Australia, Sydney, NSW Australia; 3 Centre for Clinical Epidemiology and Biostatistics, School of Medicine and Public Health, The University of Newcastle and Hunter Medical Research Institute, Newcastle, NSW Australia; 4 The Sax Institute, Sydney, NSW Australia

## Erratum

Unfortunately, after publication of this article [1], errors were noted in the second sentence of the Discussion. This sentence currently reads, “For both those with and without prior CVD in age group 45–64, major CVD incidence rates were around 50–75% higher, myocardial incidence around 250% higher and stroke incidence rates around 50–100% higher, among those of low SEP (no educational qualifications) compared to those of high SEP (university degree).” Instead, the sentence should read, “For both those with and without prior CVD in age group 45–64, major CVD incidence rates were around 50–75% higher, myocardial **infarction** incidence **rates** around **150%** higher and stroke incidence rates around 50–100% higher, among those of low SEP (no educational qualifications) compared to those of high SEP (university degree).” The changes to the sentence are in boldface for clarity.
