# Supplementary material for: Socioeconomic variation in incidence of primary and secondary major cardiovascular disease events: an Australian population-based prospective cohort study
Source: Int J Equity Health. 2016 Nov 21;15:189. doi: 10.1186/s12939-016-0471-0 (PMC5117581; doi:10.1186/s12939-016-0471-0)
Supplement: Additional file 1: Table S1. — Comparison of models for major cardiovascular disease (CVD) events rates by level of education, in those with and without prior CVD. (PDF 185 kb) [file 12939_2016_471_MOESM1_ESM.pdf]

Supplementary Table 1. Comparison of models for major cardiovascular disease (CVD) events rates by level of education, in those with and without prior CVD

	Model 1<br>HR (95% CI)	Model 2<br>HR (95% CI)	Model 3<br>HR (95% CI)
No prior CVD			
45-64 years			
No qualifications	1.62 (1.49–1.77)	1.63 (1.49–1.77)	1.59 (1.46–1.74)
Certificate/diploma/trade	1.27 (1.20–1.35)	1.27 (1.20–1.34)	1.26 (1.19–1.33)
University degree	1.00	1.00	1.00
p (test for trend)	<0.0001	<0.0001	<0.0001
65-79 years			
No qualifications	1.13 (1.04–1.23)	1.14 (1.05–1.23)	1.15 (1.05–1.25)
Certificate/diploma/trade	1.11 (1.04–1.18)	1.11 (1.04–1.18)	1.11 (1.04–1.19)
University degree	1.00	1.00	1.00
p (test for trend)	0.0032	0.0024	0.0016
≥80 years			
No qualifications	1.11 (0.99–1.25)	1.11 (0.98–1.24)	1.13 (1.00–1.27)
Certificate/diploma/trade	0.99 (0.90–1.10)	0.99 (0.90–1.09)	1.00 (0.90–1.11)
University degree	1.00	1.00	1.00
p (test for trend)	0.0402	0.0472	0.0215
Prior CVD			
45-64 years			
No qualifications	1.49 (1.34–1.65)	1.51 (1.36–1.68)	1.45 (1.30–1.62)
Certificate/diploma/trade	1.17 (1.08–1.27)	1.18 (1.09–1.28)	1.16 (1.07–1.26)
University degree	1.00	1.00	1.00

p (test for trend)	<0.0001	<0.0001	<0.0001
65-79 years			
No qualifications	1.24 (1.15–1.34)	1.24 (1.15–1.34)	1.24 (1.15–1.34)
Certificate/diploma/trade	1.17 (1.10–1.24)	1.17 (1.10–1.25)	1.17 (1.09–1.25)
University degree	1.00	1.00	1.00
p (test for trend)	<0.0001	<0.0001	<0.0001
≥80 years			
No qualifications	1.15 (1.06–1.26)	1.14 (1.04–1.24)	1.16 (1.06–1.26)
Certificate/diploma/trade	1.07 (0.99–1.15)	1.06 (0.98–1.14)	1.07 (0.99–1.15)
University degree	1.00	1.00	1.00
p (test for trend)	0.0009	0.0024	0.001

Notes. Model 1 adjusted for age and sex. Model 2 adjusted for age, sex, region of birth and region of residence. Model 3 adjusted for age, sex, region of birth, region of residence and private health insurance.
