# Supplementary material for: Socioeconomic variation in incidence of primary and secondary major cardiovascular disease events: an Australian population-based prospective cohort study
Source: Int J Equity Health. 2016 Nov 21;15:189. doi: 10.1186/s12939-016-0471-0 (PMC5117581; doi:10.1186/s12939-016-0471-0)
Supplement: Additional file 2: Table S2. — Crude rates of ischemic heart disease events and adjusted hazard ratios (HR), by education, in those with and without prior CVD. (PDF 180 kb) [file 12939_2016_471_MOESM2_ESM.pdf]

Supplementary Table 2. Crude rates of ischemic heart disease events and adjusted hazard ratios (HR), by education, in those with and without prior CVD

	No prior major CVD			*Prior major CVD*		
	**Events/py[1]**	**Crude rates[2]**	**Adjusted HR[3] (95%CI)**	**Events/py[1]**	**Crude Rates[2]**	**Adjusted HR[3] (95%CI)**
45-64 years[4]						
No qualifications	392/63395	6.18	1.74 (1.54–1.96)	322/13284	24.24	1.42 (1.23–1.65)
Cert/diploma/trade	2325/511986	4.54	1.34 (1.24–1.45)	1358/67692	20.06	1.17 (1.04–1.30)
University degree	797/239106	3.33	1.00	410/23252	17.63	1.00
p (test for trend)			<0.0001			<0.0001
65-79 years						
No qualifications	489/43220	11.31	1.14 (1.00–1.29)	831/21991	37.79	1.32 (1.19–1.48)
Cert./diploma/trade	2154/185120	11.64	1.18 (1.06–1.30)	2899/81408	35.61	1.21 (1.10–1.32)
University degree	484/46616	10.38	1.00	551/17806	30.94	1.00
p (test for trend)			0.0405			<0.0001
≥80 years						
No qualifications	260/12818	20.28	1.08 (0.90–1.31)	555/11077	50.10	1.22 (1.06–1.39)
Cert./diploma/trade	817/42637	19.16	0.97 (0.83–1.14)	1886/38783	48.63	1.15 (1.03–1.29)
University degree	181/8556	21.16	1.00	356/7932	44.88	1.00
p (test for trend)			0.3200			0.0062

Notes. 1. Events = incident hospital admission or death and py=person years of follow-up. 2. Rates are per 1000 person-years. 3. HRs adjusted for age and sex. 4. Proportional hazards assumption violated in this group with no prior CVD
