# Supplementary material for: Socioeconomic variation in incidence of primary and secondary major cardiovascular disease events: an Australian population-based prospective cohort study
Source: Int J Equity Health. 2016 Nov 21;15:189. doi: 10.1186/s12939-016-0471-0 (PMC5117581; doi:10.1186/s12939-016-0471-0)
Supplement: Additional file 3: Figure S1. — Age-adjusted rates of major cardiovascular disease (CVD) events by household income and area-level disadvantage, in those with and without prior CVD. (PDF 228 kb) [file 12939_2016_471_MOESM3_ESM.pdf]

Supplementary Figure 1. Age-adjusted rates of major cardiovascular disease (CVD) events by household income and area-level disadvantage, in those with and without prior CVD

Household income

No prior major CVD

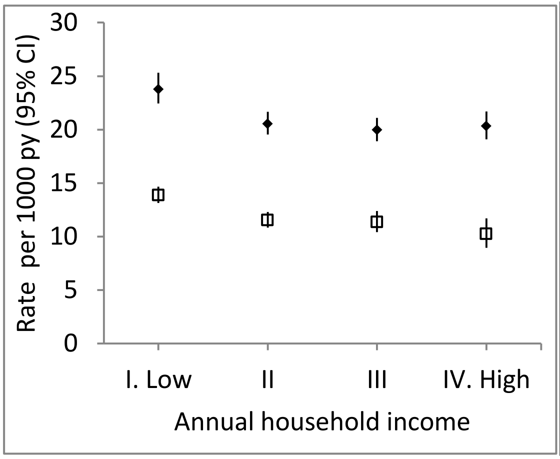

Prior major CVD

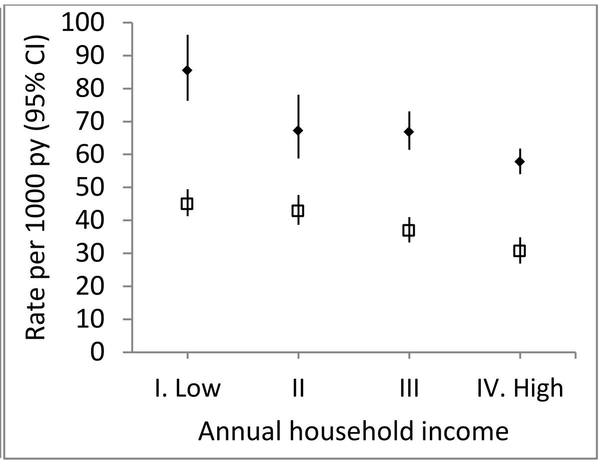

♦ Males, □ Females

Annual household income: I. < $20,000, II. $20,000-$39,999, III. $40,000-$69,999, IV.≥ $70,000

Area-level disadvantage

No prior major CVD

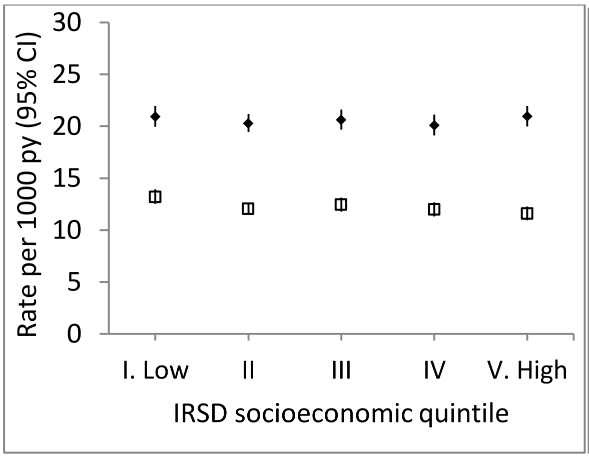

Prior major CVD

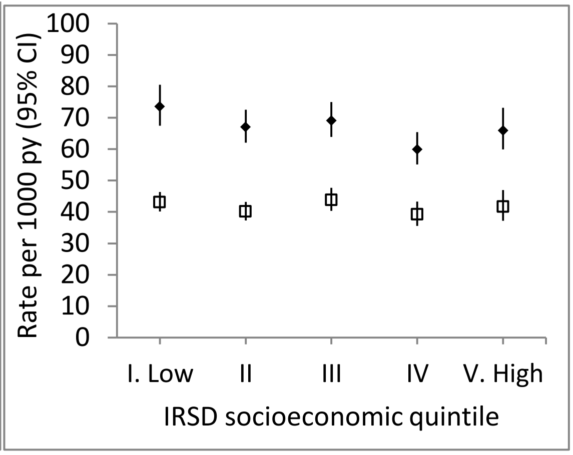

♦ Males, □ Females

IRSD=Index of Relative Socio-economic disadvantage: I. Low- most disadvantaged through V. High - least disadvantaged.
