# Supplementary material for: Socioeconomic variation in incidence of primary and secondary major cardiovascular disease events: an Australian population-based prospective cohort study
Source: Int J Equity Health. 2016 Nov 21;15:189. doi: 10.1186/s12939-016-0471-0 (PMC5117581; doi:10.1186/s12939-016-0471-0)
Supplement: Additional file 4: Table S3. — Crude rates of major cardiovascular disease (CVD) events and adjusted hazard ratios (HR) by household income and area-level disadvantage, in those with and without prior CVD. (PDF 211 kb) [file 12939_2016_471_MOESM4_ESM.pdf]

Supplementary Table 3. Crude rates of major cardiovascular disease (CVD) events and adjusted hazard ratios (HR) by household income and area-level disadvantage, in those with and without prior CVD

	No prior major CVD			*Prior major CVD*		
	Events/py[1]	Crude rates[2]	Adj. HR[3] (95%CI)	Events/py[1]	Crude rates[2]	Adj. HR[3] (95%CI)
**Household Income**						
45-64 years						
< $20,000	1014/84325	12.02	1.46 (1.35–1.57)	911/18450	49.38	1.46 (1.33–1.61)
$20,000-$39,999	1125/117673	9.56	1.16 (1.08–1.25)	707/16098	43.92	1.28 (1.16–1.41)
$40,000-$69,999	1529/178453	8.57	1.07 (1.00–1.14)	824/19848	41.52	1.19 (1.08–1.31)
≥ $70,000	2158/286096	7.54	1.00	911/25971	35.08	1.00
p (test for trend)			<0.0001			<0.0001
65-79 years						
< $20,000	2261/78767	28.70	1.11 (1.01–1.22)	3244/37779	85.87	1.16 (1.06–1.27)
$20,000-$39,999	1703/63693	26.74	1.02 (0.93–1.12)	1976/26024	75.93	0.99 (0.90–1.09)
$40,000-$69,999	950/36307	26.17	1.05 (0.94–1.16)	886/12282	72.14	0.96 (0.86–1.06)
≥ $70,000	559/21797	25.65	1.00	539/6969	77.34	1.00
p (test for trend)			0.0150			<0.0001
≥80 years						
< $20,000	1336/22448	59.52	0.98 (0.83–1.14)	2567/19064	134.66	1.15 (1.02–1.31)
$20,000-$39,999	672/11102	60.53	0.96 (0.81–1.14)	1303/9697	134.37	1.15 (1.01–1.31)
$40,000-$69,999	278/4911	56.61	0.89 (0.73–1.07)	543/3979	136.46	1.14 (0.99–1.32)
≥ $70,000	174/2644	65.80	1.00	284/2385	119.09	1.00
p (test for trend)			0.5253			0.0937
**Area–level disadvantage (IRSD[4] quintile)**						
45-64 years						

1 (Most disadvantaged)	1454/145842	9.97	1.23 (1.14–1.33)	928/20982	44.23	1.12 (1.01–1.24)
2	1767/196501	8.99	1.11 (1.04–1.20)	1069/25739	41.53	1.06 (0.96–1.17)
3	1499/163344	9.18	1.16 (1.07–1.25)	876/20409	42.92	1.09 (0.98–1.21)
4	1254/154125	8.14	1.03 (0.95–1.12)	649/17033	38.10	0.95 (0.85–1.06)
5 (Least disadvantaged)	1227/152427	8.05	1.00	602/14760	40.79	1.00
p (test for trend)			<0.0001			0.0045
65-79 years						
1 (Most disadvantaged)	1554/56742	27.39	0.97 (0.90–1.05)	2053/24125	85.10	1.02 (0.95–1.09)
2	1924/72568	26.51	0.94 (0.87–1.01)	2378/30128	78.93	0.94 (0.88–1.00)
3	1388/52081	26.65	0.95 (0.88–1.03)	1803/21916	82.27	0.98 (0.91–1.05)
4	1228/45507	26.99	0.96 (0.89–1.04)	1389/18376	75.59	0.90 (0.83–0.97)
5 (Least disadvantaged)	1213/43054	28.17	1.00	1378/16215	84.98	1.00
p (test for trend)			0.3170			0.2133
≥80 years						
1 (Most disadvantaged)	635/10952	57.98	0.94 (0.85–1.04)	1162/8171	142.20	1.06 (0.98–1.14)
2	800/14108	56.71	0.92 (0.84–1.01)	1453/10986	132.26	0.98 (0.92–1.06)
3	673/11273	59.70	0.96 (0.87–1.06)	1312/9264	141.63	1.05 (0.98–1.13)
4	703/11483	61.22	0.97 (0.88–1.07)	1315/9886	133.02	0.98 (0.91–1.06)
5 (Least disadvantaged)	879/13891	63.28	1.00	1671/12193	137.04	1.00
p (test for trend)			0.1040			0.2521

Notes. 1. Events = incident hospital admission or death and py=person years of follow-up. 2. Rates are per 1000 person-years. 3. HRs adjusted for age and sex. 4. IRSD=Index of Relative Socio-economic Disadvantage.
